# Efficacy and safety of a three-step dose escalation regimen of ropeginterferon alfa-2b in Japanese patients with polycythemia vera: a phase 3b, single-arm, multicenter study

**DOI:** 10.1007/s12185-026-04163-9

**Published:** 2026-03-09

**Authors:** Tadaaki Inano, Yuka Sugimoto, Kohshi Ohishi, Akihiko Gotoh, Tomoki Ito, Michiko Ichii, Kazuya Shimoda, Sheena Lin, Oleh Zagrijtschuk, Albert Qin, Mai Sato, Hiroaki Kawase, Toshiaki Sato, Norio Komatsu, Keita Kirito

**Affiliations:** 1 Department of Hematology, Juntendo University Graduate School of Medicine, Tokyo, Japan; 2 Department of Advanced Hematology, Juntendo University Graduate School of Medicine, Tokyo, Japan; 3 Department of Hematology and Oncology, Mie University Graduate School of Medicine, Mie, Japan; 4 Department of Transfusion Medicine and Cell Therapy, Mie University Hospital, Mie, Japan; 5 Department of Hematology, Tokyo Medical University, Tokyo, Japan; 6 First Department of Internal Medicine, Kansai Medical University, Osaka, Japan; 7 Department of Hematology and Oncology, the University of Osaka Graduate School of Medicine, Osaka, Japan; 8 Division of Hematology, Diabetes and Endocrinology, Department of Internal Medicine, Faculty of Medicine, University of Miyazaki, 5200 Kihara, Kiyotake-cho, Miyazaki City, Miyazaki Prefecture 889‑1692 Japan; 9 PharmaEssentia Corporation, Taipei, Taiwan; 10 PharmaEssentia USA Corporation, Burlington, MA USA; 11 PharmaEssentia Japan KK, Tokyo, Japan; 12 Department of Hematology and Oncology, University of Yamanashi, Yamanashi, Japan

**Keywords:** Japan, Polycythemia vera, Three-step dose escalation regimen, Ropeginterferon alfa-2b

## Abstract

Ropeginterferon alfa-2b, a monopegylated interferon α-2b, is a cytoreductive treatment for polycythemia vera (PV). In Japan, the current regimen involves titration in 50-µg increments every 2 weeks until reaching the maximum dose (500 µg), which requires considerable time. This phase 3b, open-label, single-arm, multicenter study (October 2023–July 2024) assessed the efficacy and safety of a three-step dose escalation regimen (day 1: 250 µg, week 2: 350 µg, week 4: 500 µg) of ropeginterferon alfa-2b in Japanese patients with PV (NCT06002490). Twenty-one patients were included (mean age: 57 years); most (95.2%) received ropeginterferon alfa-2b for at least 24 weeks. The complete hematologic response rate (primary endpoint; hematocrit < 45%, no phlebotomy in the previous 12 weeks, platelet count ≤ 400 × 10^9^/L, and white blood cell count ≤ 10 × 10^9^/L) was 23.8% (95% confidence interval 8.2, 47.2) at week 12 and 57.1% (34.0, 78.2) at week 24. Most patients (90.5%) reached the maximum dose of 500 µg by week 4. All patients had treatment-emergent adverse events, but none led to death, treatment discontinuation, or study withdrawal. This three-step dose escalation regimen of ropeginterferon alfa-2b has the potential to provide faster therapeutic effects in patients with PV without additional safety concerns.

**Supplementary Information:**

The online version contains supplementary material available at 10.1007/s12185-026-04163-9.

## Introduction

Polycythemia vera (PV), a Philadelphia chromosome-negative myeloproliferative neoplasm [1] that can be caused by mutations in the Janus kinase 2 (*JAK2*) gene [2], is characterized by an excessive proliferation of erythrocytes. This results in an increased risk of thrombosis [3], and, in some patients, PV can progress into myelofibrosis or acute myeloid leukemia [3]. Patients with PV can be treated with phlebotomy and low-dose aspirin [4], and for those considered to be at a higher risk of thrombosis, treatment with cytoreductive therapy, such as hydroxyurea and ropeginterferon alfa-2b, is recommended [4].

Ropeginterferon alfa-2b is a monopegylated interferon α-2b [5]. In Japan, ropeginterferon alfa-2b is initially administered as a subcutaneous injection of either 50 µg (for patients receiving other cytoreductive agents) or 100 µg, then titrated in 50-µg increments every 2 weeks up to the maximum dose (500 µg) [6]. Thus, it can take 20–24 weeks for a patient with PV to reach the maximum dose, reflecting a considerably long time to achieve a therapeutic response [5, 7]. However, safety and efficacy of a three-step escalation strategy with ropeginterferon alfa-2b was studied in China [8] and South Korea [9], and rapid hematological and molecular responses were demonstrated. Furthermore, population pharmacokinetics/pharmacodynamics and exposure response analyses of ropeginterferon alfa-2b suggested advantages of a rapid escalation strategy compared with a slower dose-escalation strategy [10].

It is currently unknown whether a three-step escalation regimen that reaches the maximum ropeginterferon alfa-2b dose earlier will achieve a more rapid therapeutic response in Japanese patients or whether this is associated with any safety concerns. Therefore, this study was conducted to assess the efficacy and safety of a three-step dose escalation regimen of ropeginterferon alfa-2b in Japanese patients with PV.

## Methods

### Study design

This study was a phase 3b, open-label, single-arm, multicenter study, conducted at six locations in Japan between October 2023 and July 2024, to assess the efficacy and safety of a three-step dose escalation regimen of ropeginterferon alfa-2b in Japanese patients with PV. The screening period was 28 days, the treatment period was 24 weeks, and there was a subsequent 14-day follow-up period.

The study was conducted in accordance with the ethical principles laid out in the Declaration of Helsinki, Good Clinical Practice regulations, and other relevant regulatory requirements. The protocol was approved by an institutional review board at each study center prior to study initiation. Written informed consent was provided by patients or their legally authorized representative, and patient anonymity was maintained by assigning unique identification numbers to all patients. The study was registered in ClinicalTrials.gov under the identifier NCT06002490.

### Patients

Patients who met the following criteria were eligible for inclusion: aged ≥ 18 years; diagnosed with PV according to the World Health Organization 2008 [11] or 2016 criteria [1]; with an inadequate response to existing therapy (hydroxyurea and/or phlebotomy) or for whom treatment with hydroxyurea and/or phlebotomy was inappropriate, per European LeukemiaNet criteria [12]; adequate hepatic function at screening (defined as total bilirubin ≤ 1.5 × the upper limit of normal [ULN], international normalized ratio of prothrombin time ≤ 1.5 × ULN, albumin > 3.5 g/dL, alanine aminotransferase ≤ 2.0 × ULN, and aspartate aminotransferase ≤ 2.0 × ULN); hemoglobin ≥ 10 g/dL (females) or ≥ 11 g/dL (males); neutrophil count ≥ 1.5 × 10^9^/L; serum creatinine ≤ 1.5 × ULN; and who agreed to use an acceptable method of birth control until 14 days following the last dose of the study drug and abstain from breastfeeding.

The following patients were excluded from the study: those with contraindications/hypersensitivity to interferon-α; previous use of interferon-α; previous use of ruxolitinib; any comorbidity that the investigator judged could impact the patient’s ability to participate; history of organ transplantation; pregnancy (at the time of enrollment or at any time during the study); participation in another clinical study within 4 weeks prior to the first dose of the study drug or unresolved sequelae from participation in a previous clinical study; symptomatic splenomegaly; circulating blasts in the peripheral blood within the last 12 weeks; or any medical condition that the investigator deemed may impact the outcome of the study or compliance with the requirements of the protocol.

### Intervention

Patients underwent a three-step dose escalation regimen of ropeginterferon alfa-2b, which was administered by subcutaneous injection via prefilled syringes into the abdomen or thigh every 2 weeks. The starting dose was 250 µg at day 1, which was increased to 350 µg at week 2, reaching the target maximum dose of 500 µg at week 4. After reaching the target dose, the dose was maintained for the duration of the treatment period and reduced to a previous dose only if safety or tolerability concerns were identified and a causal relationship with the study drug could not be ruled out.

The criteria for dose interruption were the occurrence of grade 3 or 4 adverse events (AEs) based on the Common Terminology Criteria for Adverse Events (CTCAE) version 5.0 or a neutrophil count < 0.5 × 10^9^/L. The criteria for dose reduction were the occurrence of grade 2 AEs or a neutrophil count < 0.75 × 10^9^/L.

Any patient who was receiving hydroxyurea discontinued hydroxyurea treatment on the first day of the study treatment. All patients received low-dose aspirin (between 75 and 150 mg/day) as background therapy, unless contraindicated. To reduce the risks of thromboembolic events and cardiac complications, phlebotomy was performed in patients with hematocrit ≥ 45% until it decreased to < 45%.

### Study outcomes

The primary objective was to evaluate the efficacy and safety of a three-step dose escalation regimen of ropeginterferon alfa-2b, and the secondary objectives were to determine the safety and efficacy of ropeginterferon alfa-2b.

The primary efficacy endpoint was the rate of 12-week phlebotomy-free (i.e., phlebotomy had not been required during the previous 12 weeks) complete hematologic response (CHR) at week 24. CHR was defined as the percentage of patients who met all the following criteria: hematocrit < 45% without phlebotomy during the previous 12 weeks, platelet count ≤ 400 × 10^9^/L, and white blood cell count ≤ 10 × 10^9^/L.

The secondary efficacy endpoints were the rate of CHR at week 12, the time to achieve CHR, time to first response in peripheral blood count (defined as achieving hematocrit < 45%, platelet count ≤ 400 × 10^9^/L, and white blood cell count ≤ 10 × 10^9^/L), time to achieve a 12-week phlebotomy-free interval, time to reach the response maintenance dose (defined as three consecutive identical doses), changes in hematocrit, platelet count, and white blood cell count over 12 weeks, and change from baseline at week 24 in *JAK2* V617F allele burden.

Safety endpoints included treatment-emergent AEs (TEAEs) and treatment-related TEAEs, coded using the Medical Dictionary for Regulatory Activities (MedDRA) version 27.0. Severity by grade was determined according to CTCAE version 5.0, and serious AEs were defined as any life-threatening AEs resulting in hospitalization, prolongation of hospitalization, significant disability or death, or AEs requiring medical intervention to prevent any of the outcomes listed above. Laboratory parameters, physical examination, vital signs, body weight, electrocardiography, ultrasound imaging, lung X-ray, and ophthalmological examination were also assessed.

Cardiovascular events, hemorrhagic events, thrombotic events, ocular events, psychiatric events, and immunologic reactions were considered AEs of special interest (AESIs). Events leading to a dose reduction, interruption, or study discontinuation were also recorded.

### Statistical analyses

The sample size was determined based on the results of a phase 2 study [7], assuming a CHR rate of 52% at week 24. A null CHR rate of 18% was assumed as the lower bound of the 95% confidence interval (CI) for the CHR rate at week 24 in the above study. With a two-sided type I error rate with an *α* = 0.05, a power of 90%, and a dropout rate of 15%, a sample size of 20 patients was considered reasonable.

The intent-to-treat (ITT) population was pre-specified as all patients who received ropeginterferon alfa-2b and the safety population was pre-specified as all patients who received at least one dose of ropeginterferon alfa-2b. Owing to the nature of this single-arm study, the ITT and safety populations were identical.

Categorical data were summarized using frequency (counts) and percentages with 95% CIs. Continuous data were presented by number, mean, standard deviation (SD), median, interquartile range, and CIs. Time to event data were calculated by Kaplan–Meier analyses. The primary endpoint analyses were calculated using a two-sided test with a type I error rate of 5%, using the exact method (Clopper–Pearson CIs). Missing data were not imputed, except for the primary efficacy endpoint. Patients who did not meet one or more criteria, or who discontinued treatment before meeting the response criteria, were considered non-responders for the primary efficacy endpoint.

All statistical analyses were conducted using SAS® version 9.4 or later (SAS Institute Inc, Cary, NC, USA).

## Results

### Patients

The demographics of the 21 patients included in this study are shown in Table 1. Eleven (52.4%) patients were male, and the mean ± SD age was 57.1 ± 11.9 years. All patients had an Eastern Cooperative Oncology Group Performance Status score of 0. Twelve patients had a disease duration < 3.1 years and nine had a disease duration ≥ 3.1 years (the median value reported in the long-term extension of the phase 2 trial) [13]. At baseline, 10 patients (47.6%) had a hematocrit of ≥ 45%, 17 patients (81.0%) had a platelet count > 400 × 10^9^/L, and 13 patients (61.9%) had a white blood cell count > 10 × 10^9^/L. Eleven patients had a hematocrit < 45% at baseline, and the median (interquartile range) baseline hematocrit was 44.60% (41.50–46.50%). Ten patients (47.6%) had received prior treatment with hydroxyurea. The duration of study drug treatment was > 24 weeks for most patients (95.2%), while one patient (4.8%) had a duration of 20–24 weeks.

**Table 1:** Demographic and baseline characteristics (intent-to-treat population)

Variable	Ropeginterferon alfa-2b (*N* = 21)
Sex	
Female	10 (47.6)
Male	11 (52.4)
Age, years	57.1 ± 11.9
Age group, years	
< 60	13 (61.9)
≥ 60	8 (38.1)
Risk^a^	
Low	13 (61.9)
High	8 (38.1)
Disease duration, years^b^	
< 3.1	12 (57.1)
≥ 3.1	9 (42.9)
Body weight, kg	59.56 ± 7.467
Height, cm	163.58 ± 7.353
Body mass index, kg/m^2^	22.233 ± 2.1345
ECOG Performance Status	
Grade 0	21 (100.0)
Hematocrit	
≥ 45%	10 (47.6)
< 45%	11 (52.4)
Median (IQR), %	44.60 (41.50–46.50)
Platelets	
> 400 × 10^9^/L	17 (81.0)
≤ 400 × 10^9^/L	4 (19.0)
Median (IQR), × 10^9^/L	564.0 (413.0–766.0)
White blood cells	
> 10 × 10^9^/L	13 (61.9)
≤ 10 × 10^9^/L	8 (38.1)
Median (IQR), × 10^9^/L	12.250 (7.930–17.500)
Prior hydroxyurea use	
Yes	10 (47.6)
No	11 (52.4)

^a^Low risk is defined as age < 60 years and no history of thrombosis

^b^Per median value reported in the long-term extension of the phase 2 trial [13]

Data shown as *n*, *n* (%), or mean ± standard deviation, unless otherwise indicated

Abbreviations: ECOG, Eastern Cooperative Oncology Group; IQR, interquartile range

### Primary endpoint: complete hematologic response

The CHRs at weeks 12 and 24 are shown in Table 2. At week 12, five patients achieved a CHR (23.8%; 95% CI 8.2, 47.2), and 12 patients achieved a CHR at week 24 (57.1%; 95% CI 34.0, 78.2).

**Table 2:** Complete hematologic response rate (intent-to-treat population)

Complete hematologic response rate	Patients (*N* = 21)
Week 12	5 (23.8) [8.2, 47.2]
Week 24	12 (57.1) [34.0, 78.2]

Data shown as *n* (%) [95% confidence interval]

Complete hematologic response was defined as hematocrit < 45% without phlebotomy in the previous 12 weeks, platelet count ≤ 400 × 10^9^/L, and white blood cell count ≤ 10 × 10^9^/L

### Time to response

**Fig. 1:**
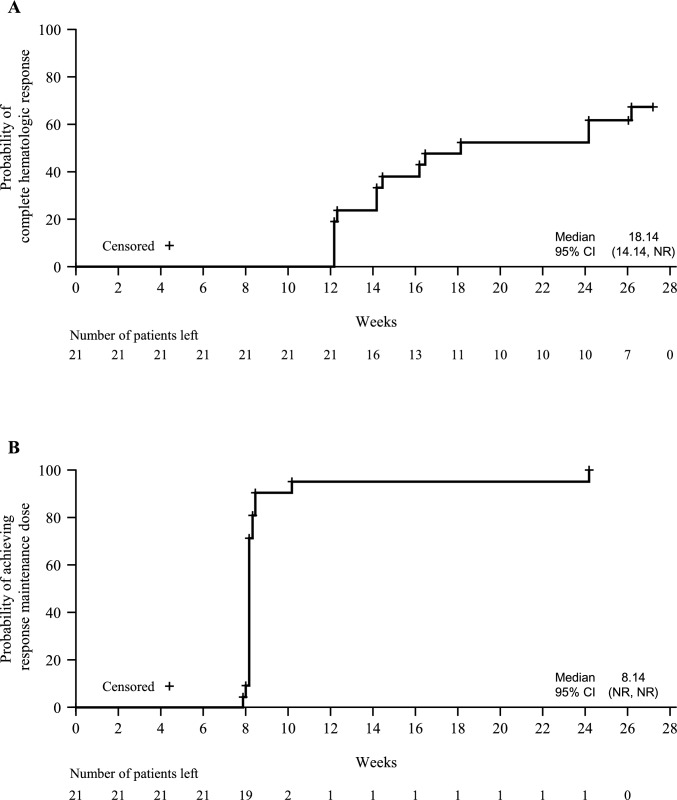
Time to **A** complete hematologic response and **B** achievement of response maintenance dose (intent-to-treat population). Complete hematologic response was defined as hematocrit < 45% with no phlebotomy in the previous 12 weeks, platelet count ≤ 400 × 10^9^/L, and white blood cell count ≤ 10 × 10^9^/L. Response maintenance dose was defined as the time of the third consecutive identical dose. CI, confidence interval; NR, not reached

The median time to CHR was 18.14 weeks (95% CI 14.14, not reached [NR]) (Fig. 1A), and the median time to reach the response maintenance dose was 8.14 weeks (95% CI NR, NR) (Fig. 1B).

**Fig. 2:**
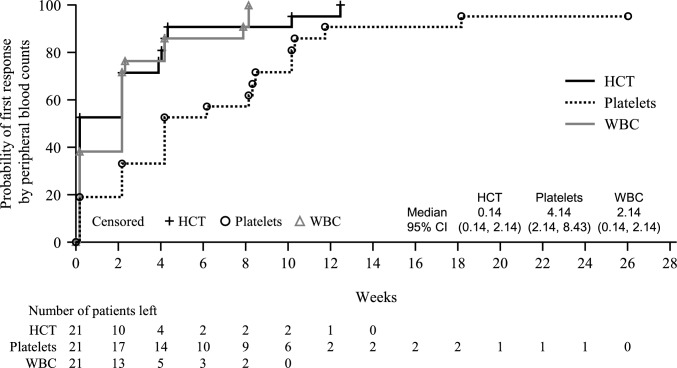
Time to first response by peripheral blood counts (intent-to-treat population). Response in peripheral blood count was defined as hematocrit < 45%, platelet count ≤ 400 × 10^9^/L, and white blood cell count ≤ 10 × 10^9^/L. CI, confidence interval; HCT, hematocrit; WBC, white blood cells

**Fig. 3:**
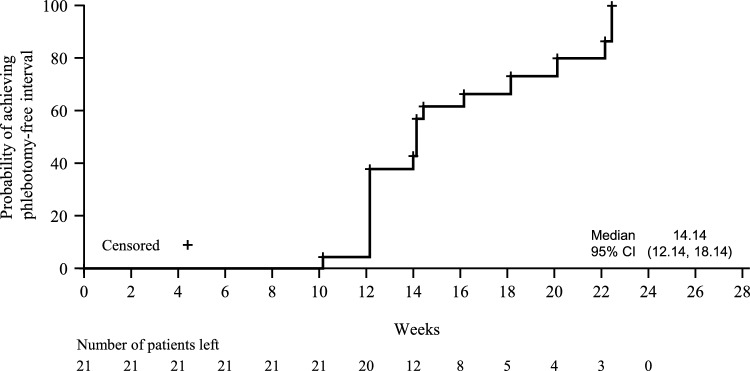
Time to achieve phlebotomy-free interval (intent-to-treat population). Phlebotomy-free interval response was defined as achieving a 12-week phlebotomy-free interval. CI, confidence interval

The median time to first response in peripheral blood count (Fig. 2) was 0.14 weeks (95% CI 0.14, 2.14) for hematocrit, 4.14 weeks (95% CI 2.14, 8.43) for platelets, and 2.14 weeks (95% CI 0.14, 2.14) for white blood cells. Hematocrit, platelet, and white blood cell levels over time are shown in Supplemental Fig. S1. The median time to achieve a 12-week phlebotomy-free interval was 14.14 weeks (95% CI 12.14, 18.14) (Fig. 3).

### Allele burden changes at week 24

**Fig. 4:**
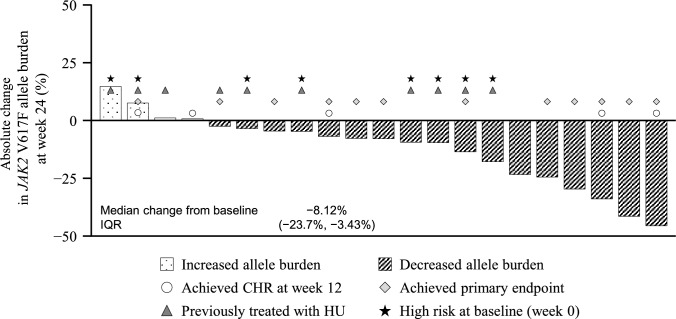
Absolute change from baseline to end of treatment in *JAK2* V617F allele burden in individual patients (intent-to-treat population). Baseline was defined as the last available measurement prior to administration of the first study treatment. CHR, complete hematologic response; HU, hydroxyurea; IQR, interquartile range; JAK, Janus kinase

The change over time in *JAK2* V617F allele burden is shown in Fig. 4, and patient-level data for allele burden at baseline and end of treatment are presented in Supplemental Table S1. Most patients (17/21; 81.0%) had a decrease in *JAK2* V617F allele burden relative to baseline. At baseline, the median (interquartile range) allele burden was 76.2% (49.7–83.2%). At the end of treatment, the median allele burden was 51.8% (34.7–79.7%), and the median change from baseline was − 8.12%. Notably, 64.7% (11/17) of patients who had a decreased *JAK2* V617F allele burden also achieved CHR at week 24, while 25.0% (1/4) of patients with an increased allele burden achieved CHR at week 24.

### Safety

At week 4, 19 (90.5%) patients were able to titrate to the maximum dose (500 µg). A summary of TEAEs is shown in Table 3, and the full list of TEAEs by MedDRA System Organ Class and Preferred Term is shown in Supplemental Table S2. Six (28.6%) patients experienced eight TEAEs that led to dose reduction; five (23.8%) patients had one dose reduction, and one (4.8%) had two dose reductions (Table 4). Five (23.8%) patients experienced seven TEAEs that led to treatment interruptions, of which two events were judged as related to the study treatment. Of the five (23.8%) patients with dose interruptions, four (19.0%) had one dose interruption, and one (4.8%) had three or more dose interruptions. The TEAEs leading to dose reductions or dose interruptions are shown in Supplemental Table S3.

**Table 3:** Summary of treatment-emergent adverse events (safety population)

Adverse event	Patients (*N* = 21)	Events
At least one TEAE	21 (100.0)	158
TEAEs leading to study withdrawal	0	0
TEAEs leading to dose reduction	6 (28.6)	8
TEAEs leading to dose interruption	5 (23.8)	7
Serious TEAEs	1 (4.8)	2
Treatment-related TEAEs	21 (100.0)	107
Treatment-related AESIs	3 (14.3)	3
Psychiatric	1 (4.8)	1
Ocular	1 (4.8)	1
Hemorrhagic	1 (4.8)	1

Data are *n* or *n* (%)

Abbreviations: AESI, adverse event of special interest; TEAE, treatment-emergent adverse event

**Table 4:** Ropeginterferon alfa-2b dose reductions and interruptions (safety population)

Dose reduction or interruption	Ropeginterferon alfa-2b (*N* = 21)
Patients with dose reductions	6 (28.6)
1 dose reduction	5 (23.8)
2 dose reductions	1 (4.8)
≥ 3 dose reductions	0
Patients with dose interruptions	5 (23.8)
1 dose interruption	4 (19.0)
2 dose interruptions	0
≥ 3 dose interruptions	1 (4.8)

Data are *n* (%)

All 21 patients experienced TEAEs, corresponding to a total of 158 events. Of these, 107 events were considered related to the study treatment (Table 3). Of the 21 patients, five (23.8%) had grade 1 (mild) TEAEs, 13 patients (61.9%) had grade 2 (moderate) TEAEs, two patients (9.5%) had grade 3 (severe) TEAEs (including pyelonephritis acute, blood triglycerides abnormal, and chronic obstructive pulmonary disease), and one patient (4.8%) had a grade 4 (life-threatening) TEAE (disseminated intravascular coagulation). Of the 26 grade 2 TEAEs, 12 were considered related to the study treatment; none of the four grade ≥ 3 TEAEs were considered to be treatment-related. One patient had two serious TEAEs, neither of which was considered related to the study treatment.

Treatment-related TEAEs by Preferred Term are shown in Table 5. The most common treatment-related TEAEs were increased urinary beta 2 microglobulin (16 patients; 76.2%) and alopecia (10 patients; 47.6%). Four (19.0%) patients experienced six AESIs; three (14.3%) patients experienced three AESIs that were judged as related to the study treatment (one psychiatric event, one ocular event, and one hemorrhagic event). No TEAEs led to death, treatment discontinuation, or withdrawal from the study.

**Table 5:** Treatment-related TEAEs (safety population)

Preferred term	Ropeginterferon alfa-2b (*N* = 21)
At least one treatment-related TEAE	21 (100.0)
Beta 2 microglobulin urine increased	16 (76.2)
Alopecia	10 (47.6)
Aspartate aminotransferase increased	6 (28.6)
Alanine aminotransferase increased	5 (23.8)
Anemia	4 (19.0)
White blood cell count decreased	4 (19.0)
Injection site reaction	3 (14.3)
Leukopenia	3 (14.3)
Blood thyroid stimulating hormone increased	2 (9.5)
Fatigue	2 (9.5)
Gamma-glutamyltransferase increased	2 (9.5)
Hepatic function abnormal	2 (9.5)
Influenza like illness	2 (9.5)
Platelet count decreased	2 (9.5)
Pyrexia	2 (9.5)
Thrombocytopenia	2 (9.5)
Thyroid function test abnormal	2 (9.5)
Abdominal discomfort	1 (4.8)
Abdominal distension	1 (4.8)
Aphthous ulcer	1 (4.8)
Arthritis	1 (4.8)
Back pain	1 (4.8)
Blood thyroid stimulating hormone decreased	1 (4.8)
Constipation	1 (4.8)
Cystitis	1 (4.8)
Depression	1 (4.8)
Diarrhea	1 (4.8)
Dry mouth	1 (4.8)
Feces soft	1 (4.8)
Liver disorder	1 (4.8)
Liver injury	1 (4.8)
Malaise	1 (4.8)
Myalgia	1 (4.8)
Neutrophil count decreased	1 (4.8)
Edema peripheral	1 (4.8)
Pleural effusion	1 (4.8)
Proteinuria	1 (4.8)
Pruritus	1 (4.8)
Rash	1 (4.8)
Retinogram abnormal	1 (4.8)
Skin hemorrhage	1 (4.8)
Stomatitis	1 (4.8)
Urticaria	1 (4.8)

Data are *n* (%)

Events were coded by Preferred Term according to the Medical Dictionary for Regulatory Activities, version 27.0

Abbreviation: TEAE, treatment-emergent adverse event

## Discussion

This study evaluated the efficacy and safety of a three-step dose escalation regimen of ropeginterferon alfa-2b in Japanese patients with PV over 24 weeks. The primary efficacy endpoint (CHR at week 24) was achieved in over half of the patients (12/21; 57.1%). By week 4, 90.5% of patients had reached the maximum ropeginterferon alfa-2b dose (500 µg). All patients experienced TEAEs, including three patients who experienced three AESIs that were related to the study drug. None of the TEAEs resulted in death, treatment discontinuation, or withdrawal from the study. Overall, this study demonstrated that the three-step dose escalation regimen of ropeginterferon alfa-2b resulted in a response in over half of the patients within 24 weeks, and no new safety concerns were identified.

In our study, the proportion of patients who achieved a CHR at week 24 (57.1%) was comparable with that reported in a previous Japanese study that used a slower titration method (i.e., 50-µg increase every 2 weeks) [7]. As previously speculated, this suggests that the three-step dose escalation regimen resulted in a similar therapeutic response over a shorter time period, which is supported by studies in other populations [14]. A study in China that evaluated the same three-step dose escalation regimen of ropeginterferon alfa-2b reported a CHR of 61.2% at week 24 [8]. Another recent study in South Korea reported a CHR of 46.0% at week 24 in patients who received the three-step dose escalation regimen [9]. Additionally, the same report indicated that achieving a CHR in a short period of time (12 weeks) is associated with achieving a molecular response at an early stage (48 weeks), suggesting that achieving a CHR in a shorter time is meaningful compared with previous dosing regimens [9]. Thus, our study provides further evidence for the faster efficacy of the three-step dose escalation regimen of ropeginterferon alfa-2b.

All measured peripheral blood parameters (hematocrit, platelets, and white blood cells) decreased over time in this study. The median time to < 45% hematocrit was 0.14 weeks; for platelets ≤ 400 × 10^9^/L, the time was 4.14 weeks, and for white blood cells ≤ 10 × 10^9^/L, 2.14 weeks. A recent observational study reported that in patients who reached the target hematocrit value (≤ 45%) but not the white blood cell target (> 12 × 10^9^/L), the risk of thrombotic events remained high (hazard ratio: 1.95 [CI 1.07, 3.55]) [15]. Considering that within approximately 4 weeks, we observed a response in all peripheral blood measures, including white blood cells, this may suggest that the three-step dose escalation regimen could reduce the risk of thrombotic events.

We observed a decrease in the *JAK2* V617F allele burden over time, with a median reduction of 8.12% at week 24 relative to baseline. A similar median reduction at week 24 relative to baseline (8.0%) was reported in a previous phase 2 study [7]. This reduction from baseline was greater at week 52 (11.6%) [7], suggesting that observation beyond 24 weeks is necessary to identify further improvements in *JAK2* V617F allele burden.

Phlebotomy can cause iron deficiency [16, 17], and it is possible for some phlebotomy-treated patients to achieve reduced hematocrit values without any improvement in PV symptoms [18]. Phlebotomy may also be less tolerated by patients, with results from a cross-sectional survey revealing that 20% of Japanese patients with PV were dissatisfied with phlebotomy treatment because of the pain of undergoing the procedure [19]. As a three-step dose escalation regimen allows patients to reach the maintenance dose sooner, this may improve patient quality of life by enabling patients to self-administer treatment earlier and reducing reliance on phlebotomy.

Although all patients in this study experienced TEAEs, most were mild or moderate in severity, and none of the severe or life-threatening TEAEs (grade ≥ 3) were judged to be related to ropeginterferon alfa-2b. No patients discontinued treatment or withdrew from the study because of TEAEs, indicating that ropeginterferon alfa-2b treatment was generally tolerable. These results are consistent with those of the earlier Japanese phase 2 study [7], and no new safety concerns were identified with the three-step dose escalation regimen. As interferon-related adverse events often occur soon after initiating treatment, it has been proposed that initiating treatment at lower doses and gradually titrating may improve tolerability [20]. The three-step dose escalation regimen in this study used a relatively high initial dose (250 μg). Nevertheless, the occurrence of TEAEs was comparable to those in previous trials [7, 14]. The recent studies in China and South Korea of the three-step dose escalation regimen also reported mostly mild or moderate TEAEs, and no grade 4 or 5 TEAEs [8, 9]. Therefore, these data suggest that initiating treatment with the 250-μg dose of ropeginterferon alfa-2b does not necessarily increase the incidence of TEAEs. Additionally, the tolerability of the three-step dose escalation regimen (250–350–500 µg over the course of 4 weeks) in Japanese patients was similar to that reported in other countries [8, 9, 14], and no additional safety concerns were identified.

We acknowledge the limitations of this study, including its open-label design and the associated risk of bias. Additionally, the duration of the study was short (24 weeks); thus, outcomes over a longer duration remain unclear. However, this short duration was chosen to focus on the tolerability of the three-step dose escalation regimen. Finally, it should be noted that the small sample size would likely have been insufficient to capture rare treatment-related TEAEs, including potentially serious or life-threatening events. Nevertheless, we consider the sample size sufficient to assess hematological and molecular efficacy.

In conclusion, this study demonstrated that the three-step dose escalation regimen (250–350–500 µg over the course of 4 weeks) of ropeginterferon alfa-2b was efficacious, as shown by hematologic response rates and reduction in *JAK2* V617F allele burden, in this cohort of Japanese patients with PV. This regimen may achieve faster therapeutic effects without additional safety concerns.

## Supplementary Information

Below is the link to the electronic supplementary material.

Supplementary file1 (PDF 201 KB)

## Data Availability

The data that support the findings of this study are available on reasonable request from the corresponding author (Kazuya Shimoda) or the sponsor, PharmaEssentia Japan KK.
