# Supplementary material for: Efficacy and safety of a three-step dose escalation regimen of ropeginterferon alfa-2b in Japanese patients with polycythemia vera: a phase 3b, single-arm, multicenter study
Source: Int J Hematol. 2026 Mar 9;123(6):848–57. doi: 10.1007/s12185-026-04163-9 (PMC13233951; doi:10.1007/s12185-026-04163-9)
Supplement: Supplementary file 1 — Supplementary file1 (PDF 201 KB) [file 12185_2026_4163_MOESM1_ESM.pdf]

Supplemental Table S1. Patient-level data for *JAK2* V617F allele burden at baseline and end of treatment

	JAK2 V617F allele burden (%)	
**Patient No.**	**Week 0 (baseline)**	**End of treatment**
1	76.236	42.253
2	94.651	69.846
3	74.281	56.097
4	77.856	92.498
5	89.494	79.673
6	83.220	83.756
7	30.624	31.581
8	49.730	40.024
9	94.809	91.380
10	15.927	13.401
11	55.759	41.963
12	95.863	90.914
13	92.662	88.125
14	23.131	15.008
15	71.528	63.417
16	80.407	34.704
17	23.743	31.199
18	47.544	40.541
19	82.167	58.479
20	54.587	13.151
21	81.496	51.787

Supplemental Table S2. All treatment-emergent adverse events (safety population)

**System Organ Class**<br>Preferred Term	**Ropeginterferon alfa-2b**<br>**(*N* = 21)**
**At least one TEAE**	21 (100.0)
**Investigations**	21 (100.0)
Beta 2 microglobulin urine increased	16 (76.2)
Aspartate aminotransferase increased	6 (28.6)
Alanine aminotransferase increased	5 (23.8)
White blood cell count decreased	4 (19.0)
Blood thyroid stimulating hormone increased	2 (9.5)
Gamma-glutamyltransferase increased	2 (9.5)
Platelet count decreased	2 (9.5)
Thyroid function test abnormal	2 (9.5)
Blood thyroid stimulating hormone decreased	1 (4.8)
Blood triglycerides abnormal	1 (4.8)
Neutrophil count decreased	1 (4.8)
Retinogram abnormal	1 (4.8)
**Skin and subcutaneous tissue disorders**	14 (66.7)
Alopecia	10 (47.6)
Pruritus	3 (14.3)
Dermatitis acneiform	1 (4.8)
Eczema	1 (4.8)
Eczema asteatotic	1 (4.8)
Rash	1 (4.8)
Skin hemorrhage	1 (4.8)
Urticaria	1 (4.8)
**Gastrointestinal disorders**	13 (61.9)
Diarrhea	3 (14.3)
Stomatitis	3 (14.3)
Constipation	2 (9.5)
Abdominal discomfort	1 (4.8)
Abdominal distension	1 (4.8)
Abdominal pain upper	1 (4.8)
Aphthous ulcer	1 (4.8)
Dry mouth	1 (4.8)
Feces soft	1 (4.8)
Toothache	1 (4.8)

System Organ Class<br>Preferred Term	Ropeginterferon alfa-2b<br>(*N* = 21)
**Blood and lymphatic system disorders**	8 (38.1)
Anemia	4 (19.0)
Leukopenia	3 (14.3)
Thrombocytopenia	2 (9.5)
Disseminated intravascular coagulation	1 (4.8)
Splenomegaly	1 (4.8)
**General disorders and administration site conditions**	8 (38.1)
Injection site reaction	3 (14.3)
Pyrexia	3 (14.3)
Fatigue	2 (9.5)
Influenza like illness	2 (9.5)
Malaise	1 (4.8)
Edema peripheral	1 (4.8)
**Infections and infestations**	7 (33.3)
Nasopharyngitis	3 (14.3)
Cystitis	2 (9.5)
Gastroenteritis	1 (4.8)
Periodontitis	1 (4.8)
Pyelonephritis acute	1 (4.8)
**Musculoskeletal and connective tissue disorders**	5 (23.8)
Arthralgia	1 (4.8)
Arthritis	1 (4.8)
Back pain	1 (4.8)
Myalgia	1 (4.8)
Pain in extremity	1 (4.8)
**Hepatobiliary disorders**	4 (19.0)
Hepatic function abnormal	2 (9.5)
Liver disorder	1 (4.8)
Liver injury	1 (4.8)
**Injury, poisoning and procedural complications**	3 (14.3)
Allergic transfusion reaction	1 (4.8)
Ligament sprain	1 (4.8)
Wound	1 (4.8)
**Metabolism and nutrition disorders**	3 (14.3)
Decreased appetite	1 (4.8)

System Organ Class<br>Preferred Term	Ropeginterferon alfa-2b<br>(*N* = 21)
Hypokalemia	1 (4.8)
Hypomagnesemia	1 (4.8)
Iron deficiency	1 (4.8)
**Respiratory, thoracic and mediastinal disorders**	3 (14.3)
Epistaxis	2 (9.5)
Chronic obstructive pulmonary disease	1 (4.8)
Pleural effusion	1 (4.8)
Rhinitis allergic	1 (4.8)
Upper respiratory tract inflammation	1 (4.8)
**Eye disorders**	2 (9.5)
Eye pain	1 (4.8)
Vision blurred	1 (4.8)
**Nervous system disorders**	2 (9.5)
Dizziness	1 (4.8)
Tension headache	1 (4.8)
**Psychiatric disorders**	2 (9.5)
Depression	1 (4.8)
Insomnia	1 (4.8)
**Renal and urinary disorders**	2 (9.5)
Dysuria	1 (4.8)
Pollakiuria	1 (4.8)
Proteinuria	1 (4.8)
**Ear and labyrinth disorders**	1 (4.8)
Vertigo	1 (4.8)
**Reproductive system and breast disorders**	1 (4.8)
Dysmenorrhea	1 (4.8)
**Vascular disorders**	1 (4.8)
Hypertension	1 (4.8)

Data are *n* (%).
Events were coded by System Organ Class and Preferred Term according to the Medical Dictionary for Regulatory Activities, version 27.0.
Abbreviation: TEAE; treatment-emergent adverse event.

Supplemental Table S3. Treatment-emergent adverse events leading to dose reductions or interruptions (safety population)

**TEAEs**	**Ropeginterferon alfa-2b** (***N*** **= 21)**
TEAEs leading to dose reduction	
Leukopenia	3 (14.3)
White blood cell count decreased	3 (14.3)
Decreased appetite	1 (4.8)
Insomnia	1 (4.8)
TEAEs leading to dose interruption	
Chronic obstructive pulmonary disease	2 (9.5)
Blood triglycerides abnormal	1 (4.8)
Depression	1 (4.8)
Disseminated intravascular coagulation	1 (4.8)
Pyelonephritis acute	1 (4.8)
Retinogram abnormal	1 (4.8)
Upper respiratory tract inflammation	1 (4.8)

Data are *n* (%).
Events were coded by Preferred Term according to the Medical Dictionary for Regulatory Activities, version 27.0.
Abbreviation: TEAE; treatment-emergent adverse event.

Supplemental Figure S1

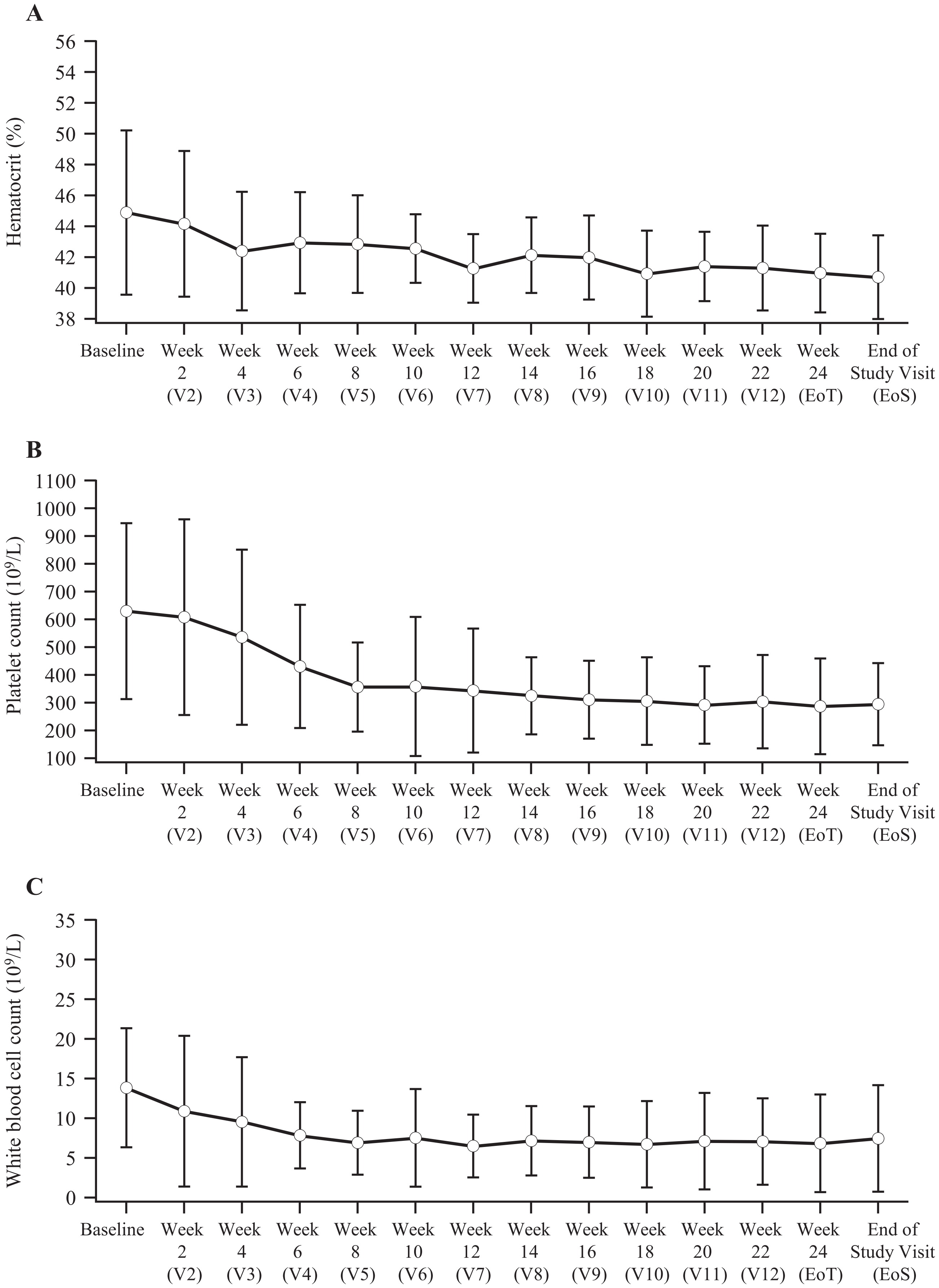

Supplemental Figure S1. Change over time in (**A**) hematocrit, (**B**) platelet count, and (**C**) white blood cell count (intent-to-treat population).

Data are mean (standard deviation).

EoS, end of study; EoT, end of treatment; V, visit.
